# Sleep Duration and Quality in Pregnant Women: A Cross-Sectional Survey in China

**DOI:** 10.3390/ijerph14070817

**Published:** 2017-07-20

**Authors:** Xianglong Xu, Dengyuan Liu, Zhangyi Zhang, Manoj Sharma, Yong Zhao

**Affiliations:** 1 School of Public Health and Management, Chongqing Medical University, Chongqing 400016, China; xianglong1989@126.com (X.X.); dengyuanliu@foxmail.com (D.L.); 2 Research Center for Medicine and Social Development, Chongqing Medical University, Chongqing 400016, China; 3 Collaborative Innovation Center of Social Risks Governance in Health, Chongqing Medical University, Chongqing 400016, China; 4 School of the Second Clinical, Chongqing Medical University, Chongqing 400016, China; ztz99c@gmail.com; 5 Department of Behavioral and Environmental Health, Jackson State University, Jackson, MS 39213, USA; manoj.sharma@jsums.edu

**Keywords:** sleep duration, quality of sleep, pregnant women, China

## Abstract

**Objectives:** Good maternal health and fetal development require sufficient and good quality of sleep during pregnancy. This study investigated sleep duration and quality in pregnant women, assessing factors with possibly influence on sleep. **Method:** A cross-sectional survey was conducted on pregnant women between June and August in 2015 in 16 hospitals in five provinces in China. A total of 2345 pregnant women aged 18 years and older were surveyed. Insufficient sleeping duration was defined as sleeping of less than 7 h per day. Excessive sleep duration was defined as sleeping of more than 9 h per day. **Results:** A total of 561 (23.9%) participants reported insufficient sleeping duration, whereas 485 (20.9%) claimed excessive sleep duration. A total of 358 (15.2%) of pregnant women reported problems regarding sleep quality. Compared to pregnant women with sufficient sleeping duration, those with insufficient sleeping duration were prone to have poor sleep quality, whereas those with excessive sleeping duration featured low possibility of poor sleep quality. High-risk groups of insufficient sleep duration include women of Han nationality, with siblings, in their first trimester of pregnancy, receiving care in low-capacity/quality hospital settings, and with daily or 1–3 days of secondhand smoke exposure. High-risk groups of excessive sleep duration include women living in rural areas, unemployed, in their third trimester of pregnancy, and receiving care in medium-capacity/quality hospital settings. High-risk groups of poor sleep quality include women of non-Han nationality, low income level, in their third trimester of pregnancy, and with insufficient sleep duration. **Conclusions:** Insufficient/excessive sleep duration and poor sleep quality commonly occur during pregnancy in China. Findings provide a better understanding of the influencing factors of insufficient/excessive sleep duration and poor quality of sleep. These findings have some implications for future interventions on sleep among pregnant women.

## 1. Introduction

Sleep problems have become an important public health issue [1], and sleep problems, such as insufficient sleep duration and poor sleep quality, also become common during pregnancy [2,3,4]. Pregnant women are easily affected by sleep disruption, and deprivation and sleep disorders [5,6]. A high prevalence of sleep disorder-related symptoms was detected in pregnant women in China [7]. Insufficient sleep duration and poor sleep quality during pregnancy may increase the risk of adverse pregnancy outcomes, including growth restriction of fetus, and postpartum depression [8,9,10,11]. Excessive sleep duration is associated with cardiovascular conditions [12]. Poor sleep quality and shorter/longer duration of sleep during pregnancy are independently associated with an increased risk of gestational diabetes [13].

Previous studies showed that age and marital status [14], low socioeconomic status [15], employment during pregnancy [16], number of pregnancies [17], trimester of pregnancy [18], passive smoking [19], and smoking and drinking [20], are associated with sleep duration among pregnant women. Previous studies showed that poor sleep quality was associated with various factors, including age [14], low socioeconomic status [15], at later stage of pregnancy [21], higher education [22], residence [7], minorities [23], and parity [24], smoking and drinking [20], sleeping duration of less than 7 h [25] among pregnant women. Poor sleep quality is also associated with rural residency among the general population [26]. To our knowledge, few studies reported influencing factors of insufficient/excessive sleep duration and their relationship with quality of sleep during pregnancy, especially among Chinese pregnant women. Previous studies found that the management of sleep disorders and poor sleep during pregnancy played important roles in health and well-being of pregnant women [22], and that all women should be screened and treated for sleep disturbances throughout pregnancy [27]. Therefore, a better understanding of duration and quality of sleeping during pregnancy will aid clinicians in improving health management and facilitating timely intervention to prevent adverse maternal and fetal outcomes among pregnant women.

The aim of the present study was to assess insufficient/excessive sleep duration, sleep quality and their influencing factors (socioeconomic characteristics, current smoking and secondhand smoke exposure, and disease conditions) among Chinese pregnant women to address the following four research objectives:

To determine the factors affecting insufficient sleep duration among Chinese pregnant women;
To identify the factors affecting excessive sleep duration among Chinese pregnant women;
To identify the factors that affect poor sleeping quality among Chinese pregnant women;
To clarify whether insufficient or excessive sleep duration poses serious consequences for sleep quality.

## 2. Materials and Methods

### 2.1. Research Methods

A previous study reported study design and methods, sampling framework, survey administration, and pilot study, population and sample, and the development of a questionnaire [28]. A cross-sectional survey was conducted among pregnant women who were admitted in 16 hospitals in five regions of Mainland China from June to August 2015. Participants included pregnant women who were examined in obstetrics clinics.

A total of 2400 women participated in the study, with a response rate of 97.76%. Among these respondents, 2345 answered all questions and were finally included in the analysis. The study was conducted in accordance with the Declaration of Helsinki, and the protocol was approved by the Ethics Committee of Chongqing Medical University (record number 2015008). All subjects provided informed consent for inclusion before participating in the study.

### 2.2. Questionnaire

#### 2.2.1. Demographic and Socio-Economic Characteristics

Demographic data include the following: residence (urban/rural); per capita monthly income of family (low income(<¥4500)/medium income (¥4500–¥9000)/high income (>¥9000), 1 USD = ¥6.79 in June 2017); age (18–25/26–35/36–45 years); occupation (rural migrant workers/urban and rural unemployed, unemployed/industrial workers of non-agricultural registered permanent residence/individual business/business services staff/civil servants/senior manager and middle-level manager in large and medium enterprises/private entrepreneur/professionals/clerks/students/others); hospital capacity/quality (high/medium/low); nationality (Han nationality/minority); without sibling (yes/no); and marital status (unmarried/marriage/remarried/divorced/widowed). Pregnancy was divided into three trimesters (first/second/third trimester of pregnancy). Educational level was categorized as low (junior middle school or below), medium (senior high school, vocational, or technical secondary school), and high (university).

Current smoking was defined as women who smoke during pregnancy and was divided into non-smokers and smokers. Frequency per week of self-reported secondhand smoke exposure was divided into none-exposure, daily, 4–6 days per week, and 1–3 days per week. Chronic hepatitis B patients were assessed with the question, “Have you ever been told by a doctor, nurse, or other health professional that you have chronic hepatitis B?” Anemia and gestational diabetes mellitus were assessed using similar questions.

For multivariable analysis for the influencing factors of insufficient/excessive sleeping and poor sleep quality, employment status was categorized as manual (rural migrant workers/industrial workers of non-agricultural registered permanent residence/business services staff), non-manual labor (individual business/civil servants/senior manager and middle-level manager in large and medium enterprise/private entrepreneur/professionals/clerk/students), unemployed, and others.

#### 2.2.2. Sleep Duration and Quality

The survey included one question about self-reported hours of sleep per day during pregnancy. “How many hours did you sleep per day?” was a closed question in which interviewees reported the number of hours of their sleep. Based on previous studies, we classified sleep hours into <7, 7–9, and >9 h per day [29,30,31]. Insufficient sleeping duration was defined as sleeping of less than 7 h. Sufficient sleeping duration was defined as sleeping between 7–9 h. Excessive sleep duration was defined as sleeping of more than 9 h. The survey included one question about self-reported quality of sleep. Participants were asked the question, “What is your quality of sleep during pregnancy?” Response options were “very good”, “good”, “average”, “poor”, and “very poor”; therefore, quality of sleep was divided into these five categories.

### 2.3. Data Analyses

Characteristics of participants were summarized using frequencies and percentages for categorical variables. We included variables with “insufficient/excessive sleeping duration and poor sleep quality” as the dependent variables in the regression model. The logistic regression model was established for the following factors affecting insufficient/excessive sleeping duration among pregnant women included the following variables: parity; nationality; with/without sibling; age; marital status; educational level; residence; per capita monthly income of the family; trimester of pregnancy; hospital capacity/quality; work; current smoking status; secondhand smoke exposure frequency per week; chronic hepatitis B condition; anemia; and gestational diabetes mellitus as the independent variables in the regression model. Ordered multivariate logistic regression was conducted for the following influencing factors of poor sleep quality among pregnant women, included the following variables: parity; nationality; with/without sibling; age; marital status; education level; residence; per capita monthly income of the family; trimester of pregnancy, hospital capacity/quality; work; current smoking; secondhand smoke exposure frequency per week; chronic hepatitis B patients; anemia; gestational diabetes mellitus; and sleeping duration as the independent variables in the regression model. All statistics were performed using two-sided tests, and statistical significance was considered at *p* < 0.05. All data analyses were performed using statistical software (SAS version 9.1.3; SAS Institute, Cary, NC, USA).

## 3. Results

### 3.1. Characteristics of Study Participants Stratified by Sleeping Duration Status

Among the 2345 participants, 1755 (74.8%) pregnant women were on their first pregnancy, and 590 (25.2%) were on their second pregnancy. The mean age was 28.1 years (SD 4.1 years), and 93 (4.0%) participants were ethnic minorities. A total of 561 (23.9%) participants reported insufficient sleeping duration, 1299 (55.4%) claimed sufficient sleep duration, and 485 (20.9%) experienced excessive sleep duration (Table 1).

### 3.2. Logistic Regression Model for Factors Influencing Insufficient and Excessive Sleep

A low possibility of insufficient sleeping duration was observed for the following pregnant women: those admitted to medium-capacity/quality hospital compared to those admitted to low-capacity/quality hospital (odds ratio (OR) = 0.33; 95% confidence interval (CI) = 0.21 to 0.54); those of non-Han nationality compared to those of Han nationality (OR = 0.45; 95% CI = 0.24 to 0.84); those without siblings compared with those with siblings (OR = 0.80; 95% CI = 0.65 to 0.97); and those in the second (OR = 0.66; 95% CI = 0.49 to 0.90) and third trimesters compared to those in the first trimester (OR = 0.65; 95% CI = 0.49 to 0.86). Pregnant women with 1–3 days secondhand smoke exposure were more likely to present insufficient sleeping duration compared to those without and daily exposure to secondhand smoke (OR = 1.43; 95% CI = 1.10 to 1.86) (Table 2).

A high probability of excessive sleeping duration was identified for the following pregnant women: those admitted to a medium capacity/quality hospital compared to those admitted to a low-capacity/quality hospital (OR = 2.57; 95% CI = 1.69 to 3.90); those who were unemployed compared to those employed (OR = 1.46; 95% CI = 1.13 to 1.88); and those in their third trimester compared to those in their first trimester (OR = 1.51; 95% CI = 1.07 to 2.13). Compared to those living in urban areas, those living in rural areas were less likely to present an excessive sleeping duration (OR = 0.64; 95% CI = 0.50 to 0.83) (Table 2).

### 3.3. Characteristics of Study Participants Stratified by Sleep Quality Status

A total of 358 (15.3%) participants reported problems in their sleep quality (13.7% poor and 1.6% very poor); 88 (14.9%) women in their second pregnancy (13.2% poor and 1.7% very poor) and 270 (15.4%) in their first pregnancy reported problems in their sleep quality (13.9% poor and 1.5% very poor); 288 (15.4%) urban women (13.7% poor and 1.7% very poor) and 70 (15.1%) rural women (13.8% poor and 1.3% very poor) reported problems in their sleep quality(Table 3).

### 3.4. Ordered Multivariate Logistic Regression Factors Influencing Poor Sleep Quality

In the ordinal logistic regression analysis model, partial regression coefficient (ß) = Estimate and OR = e^ß^. Minorities were more likely to report poor sleep quality compared to those of Han nationality (95% CI (0.01, 0.81), *p* = 0.047). Women from middle-income level families (¥4500 and ¥9000) reported that they were less likely to report poor sleep quality compared to those from low income families (<¥4500) (95% CI (−0.42, −0.01), *p* = 0.034). Pregnant women in their third trimester were more likely to report poor sleep quality compared to those in their first trimester of pregnancy (95% CI (0.04, 0.52), *p* = 0.023). Compared to pregnant women with sufficient sleeping duration, pregnant women with insufficient sleeping were more likely to report poor sleep quality (95% CI (0.01, 0.40), *p* = 0.036), whereas those with excessive sleeping were less likely to report poor sleep quality (95% CI (−0.65, −0.25), *p* < 0.000) (Table 4).

## 4. Discussion

Insufficient/excessive sleep duration and poor sleep quality are common during pregnancy. Almost one out of four pregnant women reported insufficient sleep duration, about one out of five described excessive sleep duration, and almost one out of six experienced poor sleep quality. On 29 October 2015, China abolished its One Child policy, thus allowing married couples to conceive another child. This phenomenon implies that more women in China will enter pregnancy in the future. Many women sleep insufficiently partially due to a lack of knowledge of adverse effects of this habit on their health [10]. Education on proper sleep, sleep behavior, and sleep habits help pregnant women establish their healthy sleeping awareness. Further health education and promotion measures for pregnant women are needed for better sleep hygiene practices.

Women of Han nationality, with siblings, in their first trimester of pregnancy, receiving care in low capacity/quality hospital settings, and with secondhand smoke exposure all showed an increased prevalence of insufficient sleep duration during pregnancy. Non-Han nationality members presented less chances of insufficient sleeping time. Different ethnic groups showed significant differences. A previous study in America also showed that non-Hispanic whites presented normal sleep duration [32]. China is a multi-ethnic country with 56 ethnic groups. An ethnic minority autonomous region is a place inhabited by minorities. China comprises the Tibet Autonomous Region, Xinjiang Uygur Autonomous Region, Ningxia Hui Autonomous Region, Inner Mongolia Autonomous Region, Guangxi Zhuang Autonomous Region, and five ethnic minority autonomous regions. Therefore, future programs and interventions should be tailored for each specific ethnic group. This finding contributes to a deeper understanding of insufficient sleep duration among multi-ethnic pregnant women. This study found that women in the second and third trimester of pregnancy were less likely to have insufficient sleep duration. Additionally, women in their third trimester of pregnancy showed an increased prevalence of excessive sleep duration during pregnancy. A previous study conducted in Japan showed that excessive daytime sleepiness was significantly higher in an earlier trimester of pregnancy [18]. Almost all Chinese pregnant women with jobs were on maternity leave in the third trimester of pregnancy and, thus, they were more likely to have time to sleep longer. In addition, pregnant women in their third trimester presented higher chances of poor sleep quality than those in their first trimester of pregnancy [22]; sleep in the third trimester of pregnancy is characterized by decreased sleep efficiency [27], when women in their third trimester manifested poor sleep quality, they decreased sleep efficiency and, thus, reported longer sleep duration. These may explain why women in their third trimester of pregnancy showed increased prevalence of excessive sleep duration. Thus, health education and services (for example, regarding sleeping) are vital for the whole pregnancy. This study further confirmed better sleep quality of pregnant women without siblings than those with siblings [33]. Pregnant women admitted to low capacity/quality hospitals feature insufficient sleeping time. Different levels of hospital working environment can affect work ability of medical staffs [34]. Many pregnant women who seek treatment in low-quality hospitals feature less chances of receiving adequate health-related knowledge. As a consequence, pregnant women are poorly trained with regarding to sleep hygiene measures. This study further confirmed the association of passive smoking with increased sleep disturbance during pregnancy [19]. The dose-response relationship between passive smoking and sleep disorders is not clarified and passive smoking may increase sleep disturbance through nicotine’s stimulating effect [35], nicotine withdrawal during night sleep [36], snore-related arousal [37], and the effect on pulmonary function [38]. Secondhand smoke exposure is highly prevalent among pregnant women in China (estimates range from 38.9% to 75.1%) [39]. A relatively large portion of Chinese pregnant women are affected by adverse health effects of secondhand smoke exposure. Thus, tobacco control is important and urgent in China.

Women who received care in medium-capacity/quality hospitals were also more likely to report excessive sleep duration during pregnancy. Future studies need to address the causes behind this phenomenon. Unemployment was associated with excessive sleep duration, and this result is consistent with an international literature about both pregnant women and women in the general population [40]. In the current study, 24% pregnant women were unemployed, and this value is more prevalent in China. Additionally, women living in rural areas were more likely to report excessive sleep duration, National Bureau of Statistics of China reported that 43.9% of Chinese (more than 600 million) live in rural areas in 2015 [41]. Thus, Chinese pregnant women will be disproportionately affected by adverse health effects of excessive sleep duration. Providing support for sleep health services is necessary to make them available and accessible to pregnant women who are unemployed and live in rural areas.

Women with insufficient sleep duration reported increased prevalence of poor sleep quality during pregnancy; this result agrees with those of a previous study on middle-aged and elderly Chinese adults [26]. The present study also indicated that women with excessive sleeping duration were less likely to report poor sleep quality during pregnancy. To our knowledge, most previous studies focused on consequences of insufficient sleep duration on sleep quality. However, few focused on consequences of excessive sleep duration on sleep quality. Sleep quality in this study was self-assessed, and only one question was used to evaluate sleep quality. This evaluation index is subjective and, thus, lacks objective evaluation. Adverse consequences (such as gestational diabetes and cardiovascular diseases) of excessive sleep are relatively gradual; therefore, these changes cannot be reported subjectively. This study also indicated that those women with excessive sleeping duration may report good sleep quality and continually manifest this excessive sleeping behavior. Future studies that assess sleep quality and excessive sleeping duration are needed to confirm our findings. Further studies could use combined subjective and objective methods to measure sleep quality and further probe the relationship between excessive sleep duration and poor sleep quality.

Women of non-Han nationality, of low income level, and in their third trimester of pregnancy were all more likely to report an increased prevalence of poor sleep quality during pregnancy. Pregnant women in their third trimester were more likely to report poor sleep quality than those in their first trimester of pregnancy, coinciding with a previous study among pregnant women in Taiwan [22]. A possible reason is that considering the physical changes caused by pregnancy (such as changes in body weight, thickened neck, and waistline) [7], women in their third trimester may be easily affected by these changes. During pregnancy, pregnancy-related hormones, such as progesterone, estrogen, cortisol, and oxytocin, gradually increase and, thus, may affect sleep quality in pregnant women [42]. This study showed that women from low-income level families experience poor sleep quality. General population surveys reported high risk for poor quality sleep among socio-economically disadvantaged individuals [43]. The present study indicated an association of low socioeconomic status with poorer sleep quality and fragmented sleep, coinciding with previous results among pregnant women at 10–20 weeks of gestation [25].

This study presents some limitations. First, cross-sectional survey data did not permit a reliable inference of causality. Second, face-to-face survey administration design may lead to information bias. Respondents probably did not truthfully answer the questions. Third, the study was not nationally represented. The sample consisted of pregnant women in five regions, namely, Chongqing, Chengdu, Zunyi, Liaocheng, and Tianjin, China. Chongqing, Chengdu, and Zunyi are located in South China, whereas Liaocheng and Tianjin are in North China. Fourth, although we adjusted for several variables related to socioeconomic status (age, educational level, and income), residual confounding by other factors was still possible. Fifth, this study only recruited participants during the summer months. Lack of seasonal consideration serves as a limitation owing to many life habits, including sleep change by seasons. Considering that this survey was conducted in the summer months, the weather is hot, especially in Chongqing in July and August and, thus, possibly affected sleeping duration and quality of sleep among some participants. Sixth, a shortage of data sample from minorities possibly caused deviations among minority populations. Seventh, in this study, we did not include drinking behavior, physiology of pregnancy, and depressive and anxiety symptoms. Drinking is associated with insufficient sleep duration and poor sleep quality [20]. Depressive and anxiety symptoms were associated with sleep disturbances [44]. Additionally, quality of sleep measurement was self-reported, and only one question was used to evaluate sleep quality. This evaluation index is subjective and, thus, lacks an objective evaluation.

## 5. Conclusions

Insufficient/excessive sleep duration and poor sleep quality is common during pregnancy in China. Findings provide a better understanding of the influencing factors of insufficient/excessive sleep duration and poor quality of sleep among pregnant women. Future intervention programs should focus on these high-risk groups.

## Figures and Tables

**Table 1:** Characteristics of study participants as stratified by sleep duration status in 2015 (*n*, %).

Characteristics	Insufficient Sleep Duration	Sufficient Sleep Duration	Excessive Sleep Duration
**All participants**	561 (23.9)	1299 (55.4)	485 (20.9)
**Hospital capacity/quality**			
High	471 (25.8)	1027 (56.3)	326 (17.9)
Medium	34 (10.9)	159 (51.1)	118 (37.9)
Low	56 (26.7)	113 (53.8)	41 (19.5)
**Parity**			
First pregnancy	417 (23.8)	992 (56.5)	346 (19.7)
Second pregnancy	144 (24.4)	307 (52.0)	139 (23.6)
**Age**			
18–25 years	141 (22.6)	330 (52.9)	153 (24.5)
26–35 years	383 (24.0)	905 (56.7)	307 (19.3)
36–45 years	37 (29.4)	64 (50.8)	25 (19.8)
**Nationality**			
Han nationality	549 (24.4)	1245 (55.3)	458 (20.3)
Minority	12 (12.9)	54 (58.1)	27 (29.0)
**Without sibling**			
Yes	235 (22.5)	625 (59.8)	186 (17.8)
No	326 (25.1)	674 (51.9)	299 (23.0)
**Marital status**			
Married	520 (23.6)	1227 (55.7)	458 (20.8)
Unmarried	16 (32.7)	20 (40.8)	13 (26.5)
Remarried	21 (30.0)	38 (54.3)	11 (15.7)
Divorced/Widowed	4 (19.1)	14 (66.7)	3 (14.3)
**Education level**			
Basic education	88 (21.9)	197 (49.0)	117 (29.1)
Secondary education	95 (26.8)	175 (49.4)	84 (23.7)
Higher education	378 (23.8)	927 (58.3)	284 (17.9)
**Residence**			
Urban	466 (24.8)	1067 (56.8)	347 (18.5)
Rural	95 (20.4)	232 (49.9)	138 (29.7)
**Per capita income of family**			
<¥4500	145 (23.7)	308 (50.4)	158 (25.9)
¥4500–¥9000	232 (23.5)	558 (56.4)	199 (20.1)
>¥9000	184 (24.7)	433 (58.1)	128 (17.2)
**Career**			
Rural migrant workers	20 (17.0)	61 (51.7)	37 (31.4)
Urban and rural unemployed, half of the unemployed	135 (24.4)	269 (48.6)	149 (26.9)
Industrial workers of non-agricultural registered permanent residence	10 (20.0)	33 (66.0)	7 (14.0)
Individual business	48 (24.1)	109 (54.8)	42 (21.1)
Business services staff	41 (26.5)	84 (54.2)	30 (19.4)
Civil servants	97 (24.4)	225 (56.5)	76 (19.1)
Senior manager and middle-level manager in large and medium enterprises	22 (22.9)	67 (69.8)	7 (7.3)
Private entrepreneurs	19 (21.8)	53 (60.9)	15 (17.2)
Professionals	60 (24.6)	143 (58.6)	41 (16.8)
Clerks	26 (18.7)	89 (64.0)	24 (17.3)
Students	4 (26.7)	11 (73.3)	0 (0.0)
Others	79 (27.2)	155 (53.3)	57 (19.6)
**Trimester of pregnancy**			
First trimester of pregnancy	93 (31.7)	154 (52.6)	46 (15.7)
Second trimester of pregnancy	157 (22.4)	410 (58.5)	134 (19.1)
Third trimester of pregnancy	311 (23.0)	735 (54.4)	305 (22.6)
**Current smoking**			
Non-smoker	541 (24.0)	1245 (55.2)	469 (20.80)
Smoker	20 (22.2)	54 (60.0)	16 (17.8)
**Secondhand smoke exposure frequency per week**			
None exposure	237 (20.7)	648 (56.7)	258 (22.6)
Every day	113 (27.8)	215 (52.8)	79 (19.4)
4–6 days per week	35 (22.3)	87 (55.4)	35 (22.3)
1–3 days per week	176 (27.6)	349 (54.7)	113 (17.7)
**Chronic hepatitis B patients**			
No	427 (23.7)	999 (55.5)	375 (20.8)
Yes	134 (24.6)	300 (55.2)	110 (20.2)
**Anemia**			
No	490 (23.9)	1131 (55.3)	426 (20.8)
Yes	71 (23.8)	168 (56.4)	59 (19.8)
**Gestational diabetes mellitus**			
No	533 (23.6)	1258 (55.7)	467 (20.7)
Yes	28 (32.2)	41 (47.1)	18 (20.7)

**Table 2:** Odds ratio (OR) (95% confidence interval (CI)) for factors influencing insufficient and excessive sleep in 2015.

Parameter	Insufficient Sleep			Excessive Duration		
	OR	95% CI	*p*-Value	OR	95% CI	*p*-Value
**Hospital capacity/quality**						
High vs. Low	0.99	(0.72, 1.38)	0.979	1.05	(0.72, 1.52)	0.812
Medium vs. Low	0.33	(0.21, 0.54)	<0.000	2.57	(1.69, 3.90)	<0.000
**Residence**						
Urban vs. Rural	-	-	-	0.64	(0.50, 0.83)	0.001
**Nationality**						
Minority vs. Han nationality	0.45	(0.24, 0.84)	0.012	-	-	-
**Without sibling**						
Yes vs. No	0.8	(0.65, 0.97)	0.024	-	-	-
**Education level**						
Secondary education vs. Basic education	1.32	(0.93, 1.86)	0.117	-	-	-
Higher education vs. Basic education	0.96	(0.73, 1.27)	0.797	-	-	-
**Trimester of pregnancy**						
Second trimester of pregnancy vs. First trimester of pregnancy	0.66	(0.49, 0.90)	0.009	1.08	(0.74, 1.57)	0.705
Third trimester of pregnancy vs. First trimester of pregnancy	0.65	(0.49, 0.86)	0.003	1.51	(1.07, 2.13)	0.020
**Job**						
Manual vs. Non-manual	-	-	-	1.19	(0.86, 1.64)	0.289
Unemployed vs. Non-manual	-	-	-	1.46	(1.13, 1.88)	0.003
Others vs. Non-manual	-	-	-	1.19	(0.86, 1.66)	0.296
**Secondhand smoke exposure frequency per week**						
Every day vs. None	1.43	(1.10, 1.86)	0.008	-	-	-
4–6 days per week vs. None	1.1	(0.73, 1.66)	0.637	-	-	-
1–3 days per week vs. None	1.44	(1.14, 1.80)	0.002	-	-	-
**Gestational diabetes mellitus**						
Yes vs. No	1.51	(0.94, 2.43)	0.085	-	-	-

**Table 3:** Characteristics of study participants as stratified by sleep quality status in 2015 (*n*, %).

Characteristics	Quality of Sleep				
	Very Good	Good	Average	Poor	Very Poor
**All participants**	126 (5.4)	701 (29.9)	1160 (49.4)	321 (13.7)	37 (1.6)
**Hospital capacity/quality**					
High	80 (4.4)	537 (29.4)	952 (52.2)	228 (12.5)	27 (1.5)
Medium	36 (11.6)	111(35.7)	97 (31.2)	62 (19.9)	5 (1.6)
Low	10 (4.8)	53 (25.2)	111 (52.9)	31 (14.8)	5 (2.4)
**Parity**					
First pregnancy	100 (5.7)	526 (30.0)	859 (49.0)	243 (13.9)	27 (1.5)
Second pregnancy	26 (4.4)	175 (29.7)	301 (51.0)	78 (13.2)	10 (1.7)
**Age**					
18–25 years	38 (6.1)	191 (30.6)	298 (47.8)	86 (13.8)	11 (1.8)
26–35 years	80 (5.0)	471 (29.5)	802 (50.3)	220 (13.8)	22 (1.4)
36–45 years	8 (6.4)	39 (31.0)	60 (47.6)	15 (11.9)	4 (3.2)
**Nationality**					
Han nationality	125 (5.6)	674 (29.9)	1114 (49.5)	307 (13.6)	32 (1.4)
Minority	1 (1.1)	27 (29.0)	46 (49.5)	14 (15.1)	5 (5.4)
**Without sibling**					
Yes	59 (5.6)	300 (28.7)	551 (52.7)	120 (11.5)	16 (1.5)
No	67 (5.2)	401 (30.9)	609 (46.9)	201 (15.5)	21 (1.6)
**Marital status**					
Married	118 (5.4)	660 (29.9)	1084 (49.2)	311 (14.1)	32 (1.5)
Unmarried	1 (2.0)	8 (16.3)	36 (73.5)	2 (4.1)	2 (4.1)
Remarried	5 (7.1)	25 (35.7)	31 (44.3)	8 (11.4)	1 (1.4)
Divorced/Widowed	2 (9.5)	8 (38.1)	9 (42.9)	0 (0.00)	2 (9.5)
**Education level**					
Basic education	19 (4.7)	144 (35.8)	176 (43.8)	49 (12.2)	14 (3.5)
Secondary education	27 (7.6)	111 (31.4)	170 (48.0)	42 (11.9)	4 (1.1)
Higher education	80 (5.0)	446 (28.1)	814 (51.2)	230(14.5)	19 (1.2)
**Residence**					
Urban	108 (5.7)	549 (29.2)	935 (49.7)	257 (13.7)	31 (1.7)
Rural	18 (3.9)	152 (32.7)	225 (48.4)	64 (13.8)	6 (1.3)
**Per capita monthly income of family**					
<¥4500	20 (3.3)	192 (31.4)	299 (48.9)	89 (14.6)	11 (1.8)
¥4500–¥9000	58 (5.9)	312 (31.6)	469 (47.4)	132 (13.4)	18 (1.8)
>¥9000	48 (6.4)	197 (26.4)	392 (52.6)	100 (13.4)	8 (1.1)
**Career**					
Rural migrant workers	3 (2.5)	42 (35.6)	62 (52.5)	8 (6.8)	3 (2.5)
Urban and rural unemployed, half of the unemployed	41 (7.4)	183 (33.1)	232 (42.0)	82 (14.8)	15 (2.7)
Industrial workers of non-agricultural registered permanent residence	1 (2.0)	17 (34.0)	22 (44.0)	9 (18.0)	1 (2.0)
Individual business	21 (10.6)	56 (28.1)	92 (46.2)	28 (14.1)	2 (1.0)
Business services staff	7 (4.5)	38 (24.5)	90 (58.1)	19 (12.3)	1 (0.7)
Civil servants	17 (4.3)	109 (27.4)	203 (51.0)	64 (16.1)	5 (1.3)
Senior manager and middle-level manager in large and medium enterprises	8 (8.3)	23 (24.0)	52 (54.2)	12 (12.5)	1 (1.0)
Private entrepreneur	4 (4.6)	35 (40.2)	39 (44.8)	7 (8.1)	2 (2.3)
Professionals	9 (3.7)	79 (32.4)	131 (53.7)	25 (10.3)	0 (0.0)
Clerks	2 (1.4)	33 (23.7)	86 (61.9)	16 (11.5)	2 (1.4)
Students	2 (13.3)	3 (20.0)	9 (60.0)	1 (6.7)	0 (0.0)
Others	11 (3.8)	83 (28.5)	142 (48.8)	50 (17.2)	5 (1.7)
**Trimester of pregnancy**					
First trimester of pregnancy	15 (5.1)	83 (28.3)	162 (55.3)	29 (9.9)	4 (1.4)
Second trimester of pregnancy	52 (7.4)	233 (33.2)	342 (48.8)	63 (9.0)	11 (1.6)
Third trimester of pregnancy	59 (4.4)	385 (28.5)	656 (48.6)	229 (17.0)	22 (1.6)
**Current smoking**					
Non-smoker	121 (5.4)	675 (29.9)	1115 (49.5)	308 (13.7)	36 (1.6)
Smoker	5 (5.6)	26 (28.9)	45 (50.0)	13 (14.4)	1 (1.1)
**Secondhand smoke exposure frequency per week**					
None exposure	65 (5.7)	338 (29.6)	560 (49.0)	159 (13.9)	21 (1.8)
Daily	14 (3.4)	136 (33.4)	211 (51.8)	43 (10.6)	3 (0.7)
4–6 days per week	6 (3.8)	40 (25.5)	79 (50.3)	29 (18.5)	3 (1.9)
1–3 days per week	41 (6.4)	187(29.3)	310 (48.6)	90 (14.1)	10 (1.6)
**Chronic hepatitis B patients**					
No	93 (5.16)	561 (31.2)	890 (49.4)	231 (12.8)	26 (1.4)
Yes	33 (6.1)	140 (25.7)	270 (49.6)	90 (16.5)	11 (2.0)
**Anemia**					
No	113 (5.5)	616 (30.1)	1017 (49.7)	271 (13.2)	30 (1.5)
Yes	13 (4.4)	85 (28.5)	143 (48.0)	50 (16.8)	7 (2.4)
**Gestational diabetes mellitus**					
No	119 (5.3)	681 (30.2)	1117 (49.5)	304 (13.5)	37 (1.6)
Yes	7 (8.1)	20 (23.0)	43 (49.4)	17 (19.5)	0 (0.00)

**Table 4:** Ordered multivariate logistic regression for poor quality of sleep among pregnant women in 2015.

Parameter	Category	Estimate	Standard Error	95% CI		*p*-Value
**Nationality**	Minority	0.41	0.20	0.01	0.81	0.047
	Han nationality (ref.)					
**Age**	26–35 years	−0.02	0.10	−0.21	0.17	0.816
	36–45 years	−0.15	0.20	−0.54	0.25	0.468
	18–25 years (ref.)					
**Without sibling**	Yes	−0.07	0.08	−0.23	0.09	0.398
	No (ref.)					
**Marital status**	Unmarried	0.41	0.26	−0.11	0.93	0.123
	Remarried	−0.28	0.24	−0.74	0.19	0.243
	Divorced or Widowed	−0.58	0.43	−1.42	0.26	0.173
	Married (ref.)					
**Education level**	Secondary education	−0.05	0.1	−0.33	0.22	0.707
	Higher education	0.20	0.13	−0.05	0.45	0.121
	Basic education (ref.)					
**Residence**	Urban	−0.07	0.11	−0.29	0.14	0.497
	Rural (ref.)					
**Per capita month income of family**	¥4500–¥9000	−0.22	0.10	−0.42	−0.01	0.034
	>¥9000	−0.19	0.11	−0.41	0.03	0.092
	<¥4500 (ref.)					
**Job**	Manual	0.12	0.13	−0.12	0.37	0.329
	Unemployed	−0.07	0.11	−0.28	0.15	0.539
	Others	0.21	0.13	−0.04	0.46	0.097
	Non-manual (ref.)					
**Number of pregnancy**	Secondpregnancy	0.14	0.10	−0.05	0.34	0.142
	First pregnancy (ref.)					
**Trimester of pregnancy**	Second trimester	−0.17	0.13	−0.43	0.09	0.199
	Third trimester	0.28	0.12	0.04	0.52	0.023
	First trimester (ref.)					
**Hospital capacity/quality**	High	−0.14	0.14	−0.42	0.13	0.308
	Medium	−0.33	0.18	−0.68	0.02	0.066
	Low (ref.)					
**Current smoking**	Smoker	0.01	0.20	−0.40	0.40	0.998
	Non-smoker (ref.)					
**Secondhand smoke exposure frequency per week**	Every day	−0.13	0.11	−0.34	0.08	0.231
	4–6 days per week	0.32	0.16	−0.01	0.64	0.053
	1–3 days per week	−0.05	0.09	−0.24	0.13	0.566
	None (ref.)					
**Chronic hepatitis B patients**	Yes	0.24	0.15	−0.06	0.53	0.119
	No (ref.)					
**Anemia**	Yes	−0.05	0.18	−0.39	0.30	0.797
	No (ref.)					
**Gestational diabetes mellitus**	Yes	−0.16	0.25	−0.64	0.33	0.521
	No (ref.)					
**Sleep duration**	Insufficient sleeping duration	0.21	0.10	0.01	0.40	0.036
	Excessive sleep duration	−0.45	0.10	−0.65	−0.25	<0.000
	Sufficient sleep duration (ref.)					

## References

[1] Colten H.R., Altevogt B.M. (2006). Congressional language establishing the national center on sleep disorders research, § 285b-7—Sleep disorders and sleep deprivation. Sleep Disorders and Sleep Deprivation: An Unmet Public Health Problem.

[2] Hutchison B.L., Stone P.R., Mccowan L.M., Stewart A.W., Thompson J.M., Mitchell E.A. (2012). A postal survey of maternal sleep in late pregnancy. BMC Pregnancy Childbirth.

[3] Naud K., Ouellet A., Brown C., Pasquier J.C., Moutquin J.M. (2010). Is sleep disturbed in pregnancy?. J. Obstet. Gynaecol. Can..

[4] Palagini L., Gemignani A., Banti S., Manconi M., Mauri M., Riemann D. (2014). Chronic sleep loss during pregnancy as a determinant of stress: Impact on pregnancy outcome. Sleep Med..

[5] Wojtyniak M., Szot K., Wrzalik R., Rodenbucher C., Roth G., Waser R. (2003). The prevalence of restless legs syndrome among pregnant women in japan and the relationship between restless legs syndrome and sleep problems. Sleep.

[6] Grigsbytoussaint D.S., Turi K.N., Krupa M., Williams N.J., Pandiperumal S.R., Jeanlouis G. (2015). Sleep insufficiency and the natural environment: Results from the US behavioral risk factor surveillance system survey. Prev. Med..

[7] Cai X.H., Xie Y.P., Li X.C., Qu W.L., Li T., Wang H.X., Lv J.Q., Wang L.X. (2013). The prevalence and associated risk factors of sleep disorder-related symptoms in pregnant women in China. Sleep Breath..

[8] Tsai S.Y., Lee P.L., Lin J.W., Lee C.N. (2016). Cross-sectional and longitudinal associations between sleep and health-related quality of life in pregnant women: A prospective observational study. Int. J. Nurs. Stud..

[9] Bourjeily G., Raker C.A., Chalhoub M., Miller M.A. (2010). Pregnancy and fetal outcomes of symptoms of sleep-disordered breathing. Eur. Respir. J..

[10] Chang J.J., Pien G.W., Duntley S.P., Macones G.A. (2010). Sleep deprivation during pregnancy and maternal and fetal outcomes: Is there a relationship?. Sleep Med. Rev..

[11] Fung A.M., Wilson D.L., Lappas M., Howard M., Barnes M., O’Donoghue F., Tong S., Esdale H., Fleming G., Walker S.P. (2013). Effects of maternal obstructive sleep apnoea on fetal growth: A prospective cohort study. PLoS ONE.

[12] Kim Y., Wilkens L.R., Schembre S.M., Henderson B.E., Kolonel L.N., Goodman M.T. (2013). Insufficient and excessive amounts of sleep increase the risk of premature death from cardiovascular and other diseases: The multiethnic cohort study. Prev. Med..

[13] Wang H., Leng J., Li W., Wang L., Zhang C., Li W., Liu H., Zhang S., Chan J., Hu G. (2016). Sleep duration and quality, and risk of gestational diabetes mellitus in pregnant Chinese women. Diabet. Med..

[14] Duke C.H., Williamson J.A., Snook K.R., Finch K.C., Sullivan K.L. (2017). Association between fruit and vegetable consumption and sleep quantity in pregnant women. Matern. Child Health J..

[15] Okun M.L., Tolge M., Hall M. (2014). Low socioeconomic status negatively affects sleep in pregnant women. J. Obstet. Gynecol. Neonatal Nurs..

[16] Facco F.L., Kramer J., Ho K.H., Zee P.C., Grobman W.A. (2010). Sleep disturbances in pregnancy. Obstet. Gynecol..

[17] Lee K.A., Zaffke M.E., Mcenany G. (2000). Parity and sleep patterns during and after pregnancy. Obstet. Gynecol..

[18] Nakagome S., Kaneita Y., Itani O., Ikeda M., Ichinose A., Morioka H., Osaki Y., Ohida T. (2014). Excessive daytime sleepiness among pregnant women: An epidemiological study. Sleep Biol. Rhythm..

[19] Ohida T., Kaneita Y., Osaki Y., Harano S., Tanihata T., Takemura S., Wada K., Kanda H., Hayashi K., Uchiyama M. (2007). Is passive smoking associated with sleep disturbance among pregnant women?. Sleep.

[20] Kaneita Y., Ohida T., Takemura S., Sone T., Suzuki K., Miyake T., Yokoyama E., Umeda T. (2005). Relation of smoking and drinking to sleep disturbance among Japanese pregnant women. Prev. Med..

[21] Cheng C.Y., Wang P., Liou S.R. Sleep quality and its related factors during pregnancy. Proceedings of the 24th International Nursing Research Congress.

[22] Shobeiri F., Ebrahimi R., Khodakarami B., Roshanei G. (2016). Sleep quality and its predictive factors in nulliparous pregnant women. Res. J. Pharm. Biol. Chem. Sci..

[23] Rawal S., Hinkle S.N., Zhu Y., Albert P.S., Zhang C. (2017). A longitudinal study of sleep duration in pregnancy and subsequent risk of gestational diabetes: Findings from a prospective, multiracial cohort. Am. J. Obstet. Gynecol..

[24] Naghi I., Keypour F., Ahari S.B., Tavalai S.A., Khak M. (2011). Sleep disturbance in late pregnancy and type and duration of labour. J. Obstet..

[25] Suzuki K., Ohida T., Sone T., Takemura S., Yokoyama E., Miyake T., Harano S., Nozaki N., Motojima S., Suga M. (2003). An epidemiological study of sleep problems among the Japanese pregnant women. Nihon Kōshū Eisei Zasshi.

[26] Haseli-Mashhadi N., Dadd T., An P., Yu Z., Xu L., Franco O.H. (2009). Sleep quality in middle-aged and elderly chinese: Distribution, associated factors and associations with cardio-metabolic risk factors. BMC Public Health.

[27] Mindell J.A., Cook R.A., Nikolovski J. (2015). Sleep patterns and sleep disturbances across pregnancy. Sleep Med..

[28] Wang L., Xu X., Baker P., Chao T., Lei Z., Qi H., Yong Z. (2016). Patterns and associated factors of caesarean delivery intention among expectant mothers in China: Implications from the implementation of China’s new national two-child policy. Int. J. Environ. Res. Public Health.

[29] Watson N.F., Badr M.S., Belenky G., Bliwise D.L., Buxton O.M., Buysse D., Dinges D.F., Gangwisch J., Grandner M.A., Kushida C. (2015). Joint consensus statement of the american academy of sleep medicine and sleep research society on the recommended amount of sleep for a healthy adult: Methodology and discussion. J. Clin. Sleep Med..

[30] Petrov M.E., Lichstein K.L. (2015). Differences in sleep between black and white adults: An update and future directions. Sleep Med..

[31] Pergola B.L., Moonie S., Pharr J., Bungum T., Anderson J.L. (2017). Sleep duration associated with cardiovascular conditions among adult Nevadans. Sleep Med..

[32] Borodulin K., Evenson K.R., Monda K., Wen F., Herring A.H., Dole N. (2010). Physical activity and sleep among pregnant women. Paediatr. Perinat. Epidemiol..

[33] Vermeesch A.L., Stommel M. (2013). Physical activity and acculturation among U.S. Latinas of childbearing age. West. J. Nurs. Res..

[34] Gimeno D., Felknor S., Burau K.D., Delclos G.L. (2005). Organisational and occupational risk factors associated with work related injuries among public hospital employees in Costa Rica. Occup. Environ. Med..

[35] Guzmán-Marín R., Alam M.N., Mihailescu S., Szymusiak R., Mcginty D., Drucker-Colín R. (2001). Subcutaneous administration of nicotine changes dorsal raphe serotonergic neurons discharge rate during rem sleep. Brain Res..

[36] Moreno-Coutino A., Calderon-Ezquerro C., Drucker-Colin R. (2007). Long-term changes in sleep and depressive symptoms of smokers in abstinence. Nicotine Tob. Res..

[37] Montgomerydowns H.E., Gozal D. (2006). Snore-associated sleep fragmentation in infancy: Mental development effects and contribution of secondhand cigarette smoke exposure. Pediatrics.

[38] Argacha J.F., Xhaët O., Gujic M., Adamopoulos D., Beloka S., Dreyfuss C., Degaute J.P., van de Borne P. (2008). Nicotine increases chemoreflex sensitivity to hypoxia in non-smokers. J. Hypertens..

[39] Zhang L., Hsia J., Tu X., Xia Y., Zhang L., Bi Z., Liu H., Li X., Stanton B. (2015). Peer reviewed: Exposure to secondhand tobacco smoke and interventions among pregnant women in china: A systematic review. Prev. Chronic Dis..

[40] Signal T.L., Paine S.J., Sweeney B., Priston M., Muller D., Smith A., Lee K.A., Huthwaite M., Reid P., Gander P. (2014). Prevalence of abnormal sleep duration and excessive daytime sleepiness in pregnancy and the role of socio-demographic factors: Comparing pregnant women with women in the general population. Sleep Med..

[41] National Bureau of Statistics of China Population of China in 2015. http://data.stats.gov.cn/easyquery.htm?cn=C01&zb=A0301&sj=2015.

[42] Mehta N., Shafi F., Bhat A. (2015). Unique aspects of sleep in women. MO. Med..

[43] Tsai S.Y., Lin J.W., Kuo L.T., Thomas K.A. (2012). Daily sleep and fatigue characteristics in nulliparous women during the third trimester of pregnancy. Sleep.

[44] Polo-Kantola P., Aukia L., Karlsson H., Karlsson L., Paavonen E.J. (2017). Sleep quality during pregnancy: Associations with depressive and anxiety symptoms. Acta Obstet. Gynecol. Scand..

